# Supplementary material for: Unveiling RNA structure-mediated regulations of RNA stability in wheat
Source: Nat Commun. 2024 Nov 20;15:10042. doi: 10.1038/s41467-024-54172-7 (PMC11579497; doi:10.1038/s41467-024-54172-7)
Supplement: Supplementary file 1 — Supplementary Information [file 41467_2024_54172_MOESM1_ESM.pdf]

Supplementary Information for

Unveiling RNA Structure-mediated Regulations of RNA Stability in Wheat

Haidan Wu[1†], Haopeng Yu[1,2†], Yueying Zhang[1,2†], Bibo Yang[2†], Wenqing Sun[1], Lanying Ren[1], Yuchen Li[1], Qianqian Li[2,3], Bao Liu[1*], Yiliang Ding[2*], Huakun Zhang[1*]

[1]Key Laboratory of Molecular Epigenetics of the Ministry of Education, Northeast Normal University, Changchun 130024, China

[2]Department of Cell and Developmental Biology, John Innes Centre, Norwich Research Park, Norwich, NR4 7UH, United Kingdom

[3]Guangdong Provincial Key Laboratory of Applied Botany & Key Laboratory of South China Agricultural Plant Molecular Analysis and Genetic Improvement, South China Botanical Garden, Chinese Academy of Sciences, Guangzhou 510650, China

†These authors contributed equally

*Correspondence: zhanghk045@nenu.edu.cn or yiliang.ding@jic.ac.uk or baoliu@nenu.edu.cn

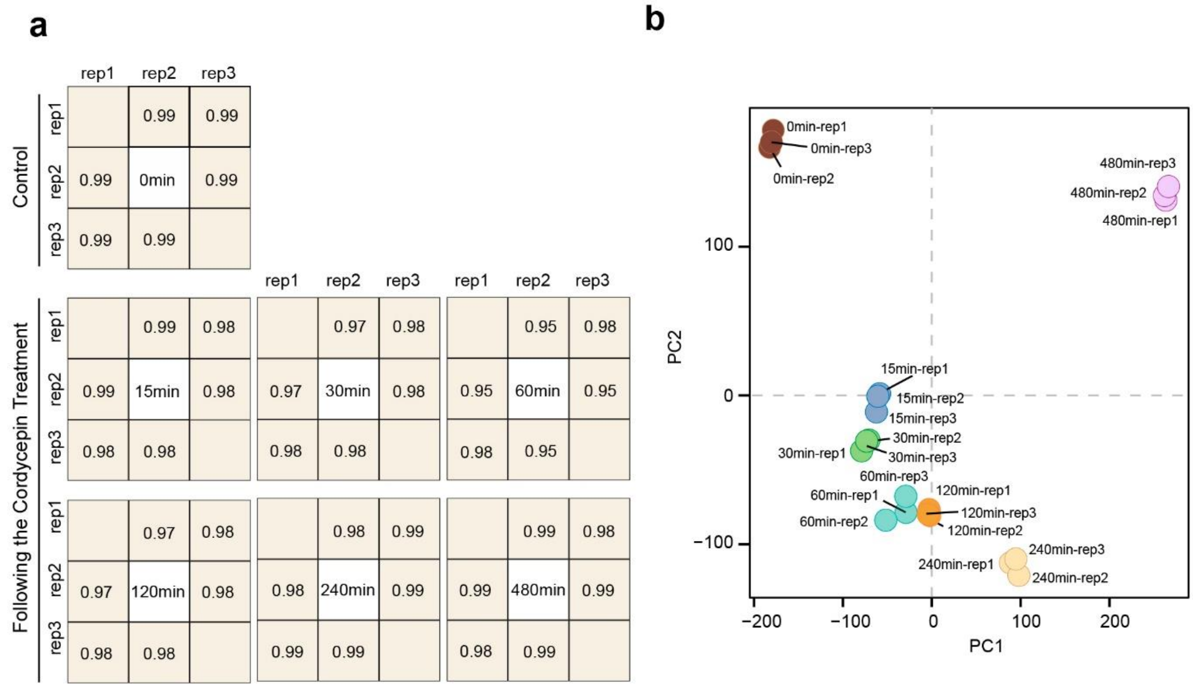

Supplementary Fig. 1 | Wheat mRNA decay data overview. a, Correlation coefficients of RNA abundances in the three biological replicates for each time point (two-sided Pearson correlation test). **b,** The PCA plot illustrates the clustering of RNA abundances in the three biological replicates for each time point.

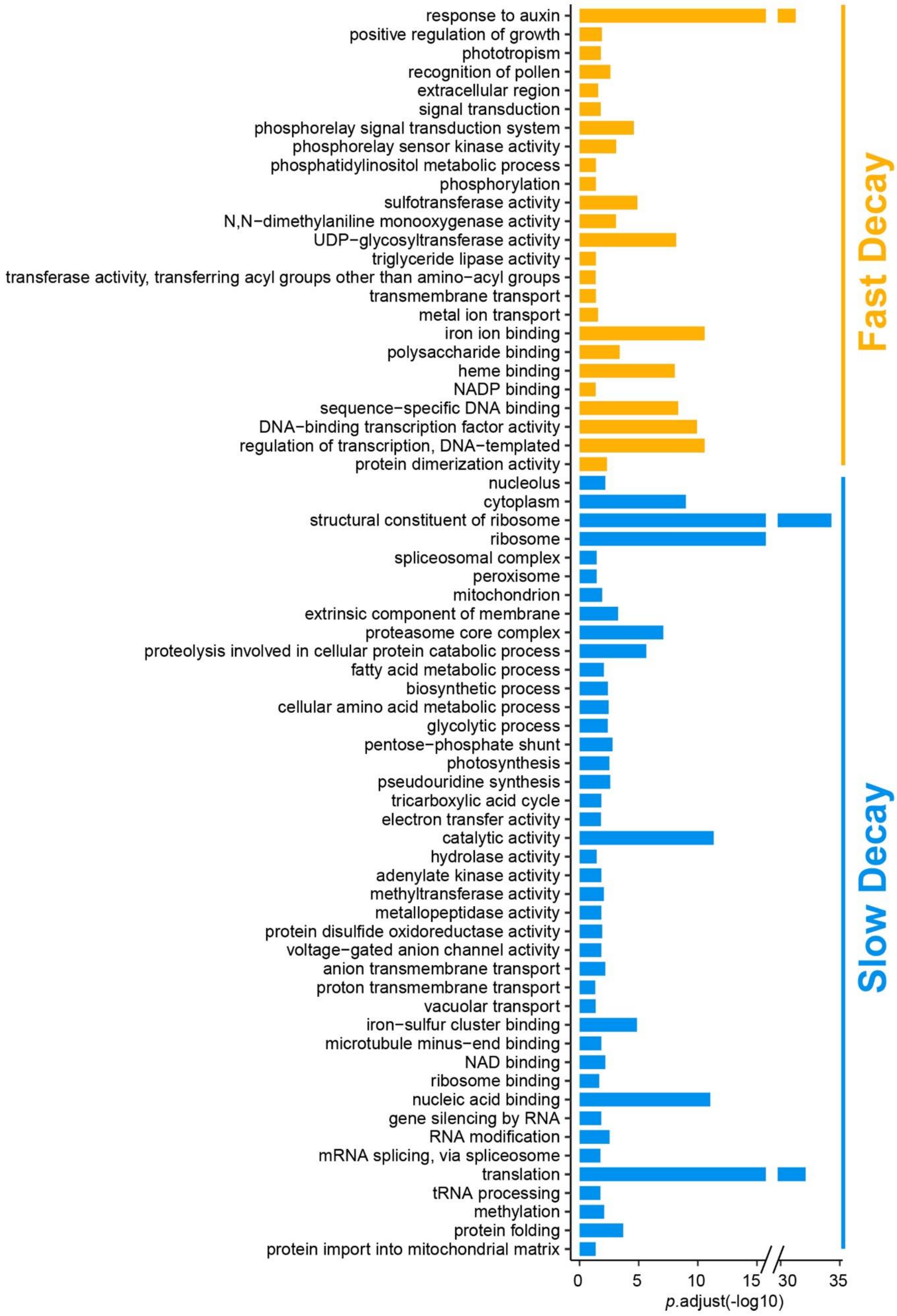

Supplementary Fig. 2 | Gene ontology (GO) analysis for the genes with the fastest and slowest decay rates. GO enrichments for the 10% genes with the fastest decay rates (n=3,343, orange) and the 10% genes with slowest decay rates (n=3,343, blue).

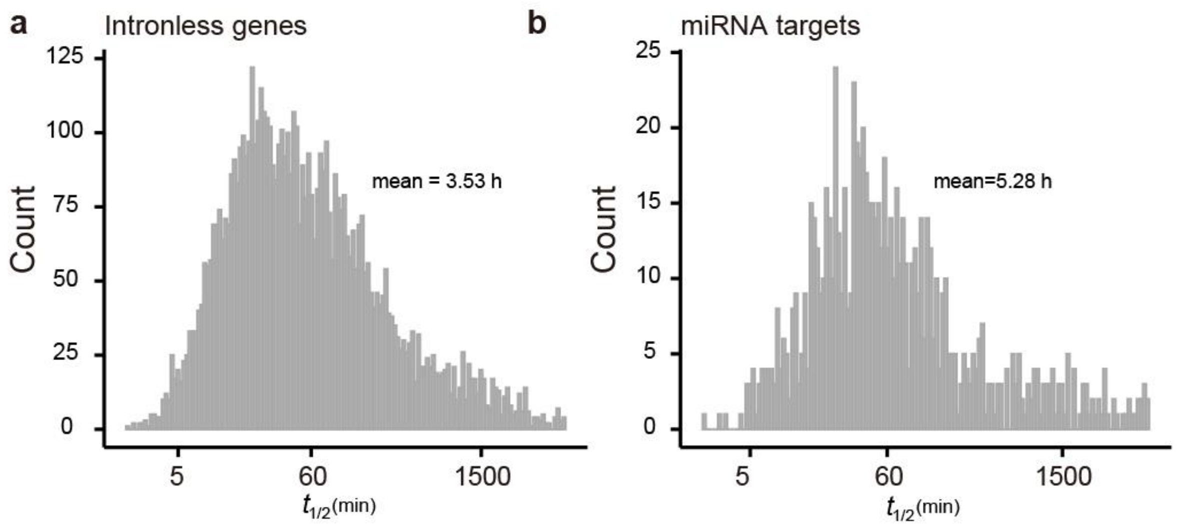

Supplementary Fig. 3 | The distributions of mRNA half-lives for intronless genes and miRNA target genes. a-b, The average half-lives of intronless genes and miRNA-targeted mRNAs are 3.53 hours and 5.28 hours, respectively.

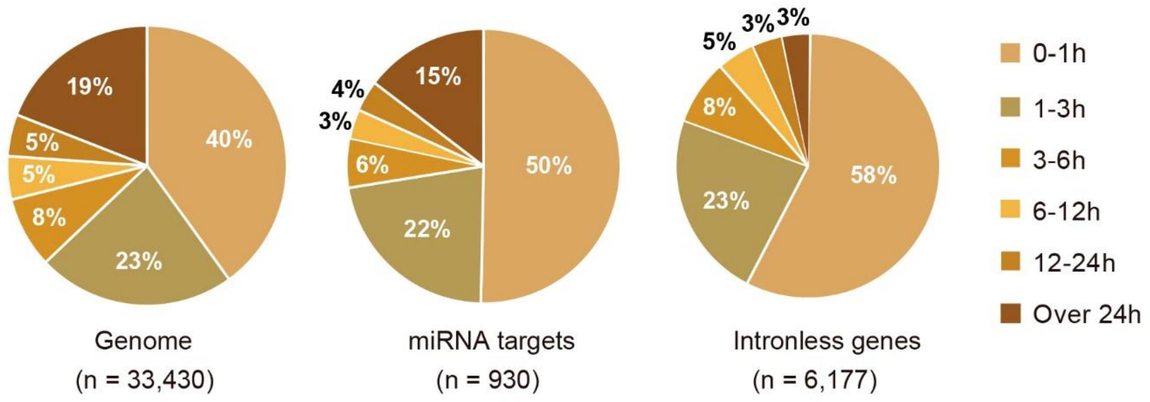

Supplementary Fig. 4 | The distribution patterns of half-lives vary among the three subgroups. The gene counts are 33,430 for genome-wide genes, 930 for miRNA targets, and 6,177 for intronless genes, respectively.

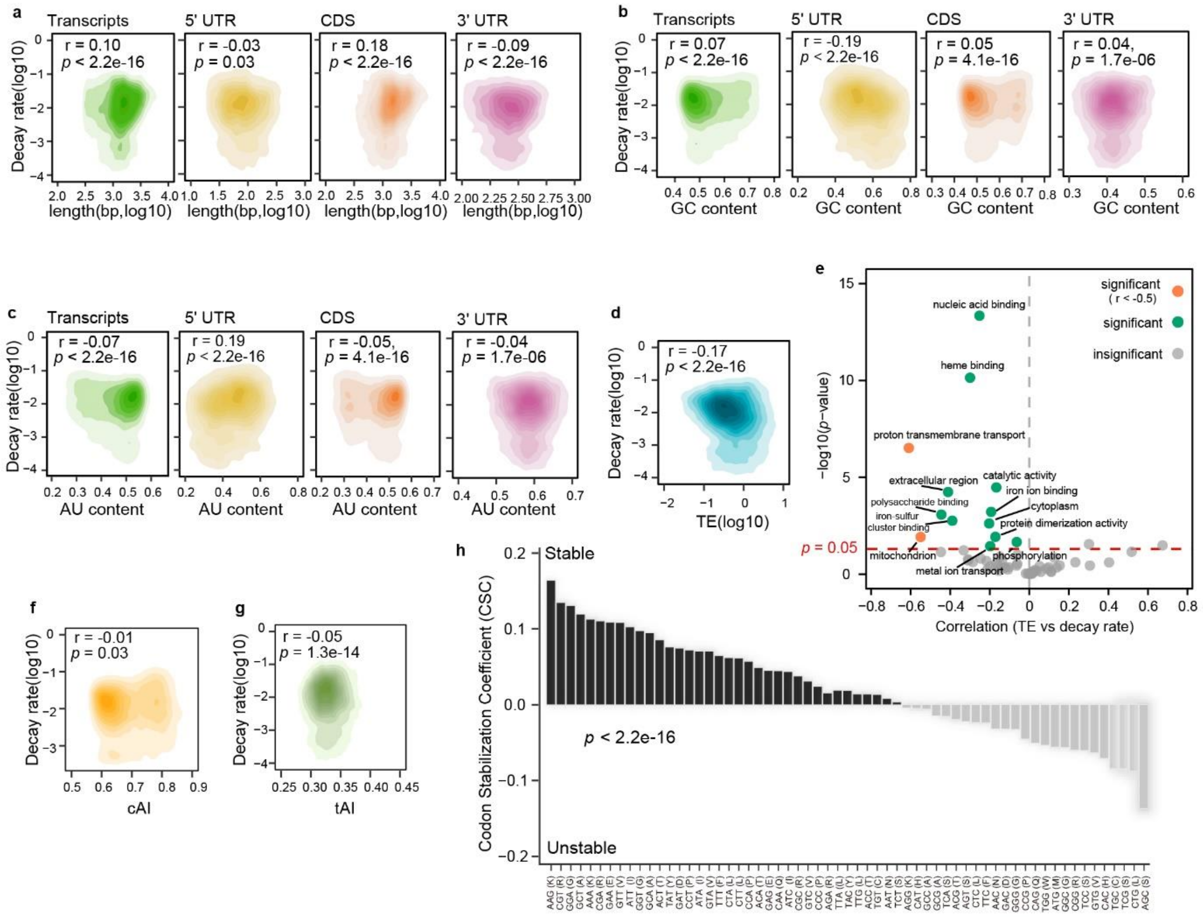

Supplementary Fig. 5 | mRNA features may contribute to mRNA decay in tetraploid wheat. a-c, The correlations of sequence length (**a**), GC content (**b**) and AU content (**c**) with mRNA decay rates in different genetic regions at the genome-wide level (two-sided Pearson correlation test). **d,** The correlations analysis between translation efficiency and RNA decay rates, **e,** Correlation analysis between translation efficiency and RNA decay rate across gene clusters (significant, $p < 0.05$, two-sided Pearson correlation test). **f-g,** The correlations of cAI (**f**) and tAI (**g**) with mRNA decay rates (two-sided Pearson correlation test). **h,** The Codon Stabilization Coefficient (CSC) is calculated for each codon to measure the correlation between codon occurrences and mRNA decay rates ($p < 2.2e\text{-}16$, two-sided Pearson correlation test).

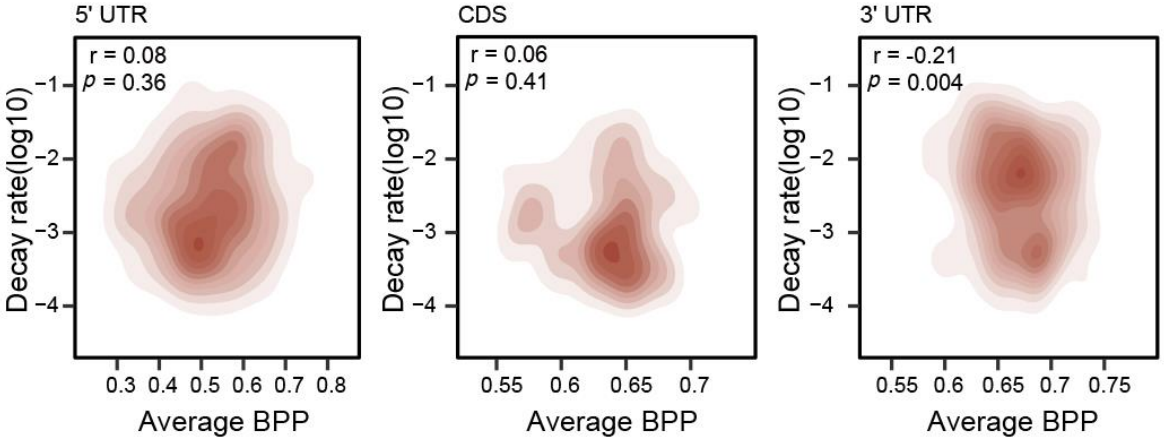

Supplementary Fig. 6 | Correlations between mRNA decay rate and average base pairing probability (BPP) in different genetic regions. The correlations between mRNA decay rate and the mean BPP in the 5' UTR, CDS and 3' UTR (5' UTR, $r = 0.08$, $p = 0.36$; CDS, $r = 0.06$, $p = 0.41$, 3' UTR, $r = -0.21$, $p = 0.004$, two-sided Pearson correlation test).

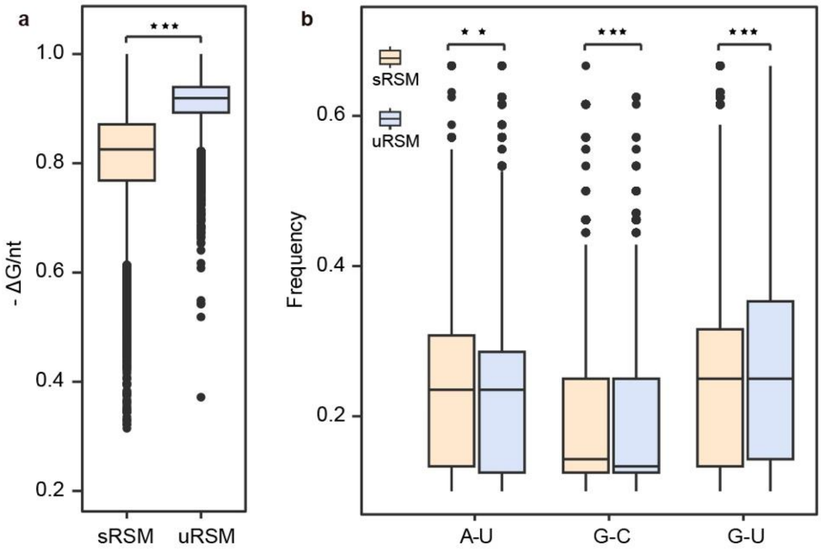

Supplementary Fig. 7 | The structural features between stable and unstable RNA structural motifs (sRSMs and uRSMs). a, The length-normalized minimum thermodynamic free energy (-ΔG/nt) of sRSMs are significantly lower than those of uRSMs (***$p < 0.001$, one-sided Student's *t*-test). **b,** Among the three base-pair types (A-U, G-C and G-U), sRSMs also contain significantly more canonical base pairs (A-U and G-C) and less non-canonical base pairs (G-U) than uRSMs (**$p < 0.01$, ***$p < 0.001$, one-sided Student's *t*-test).

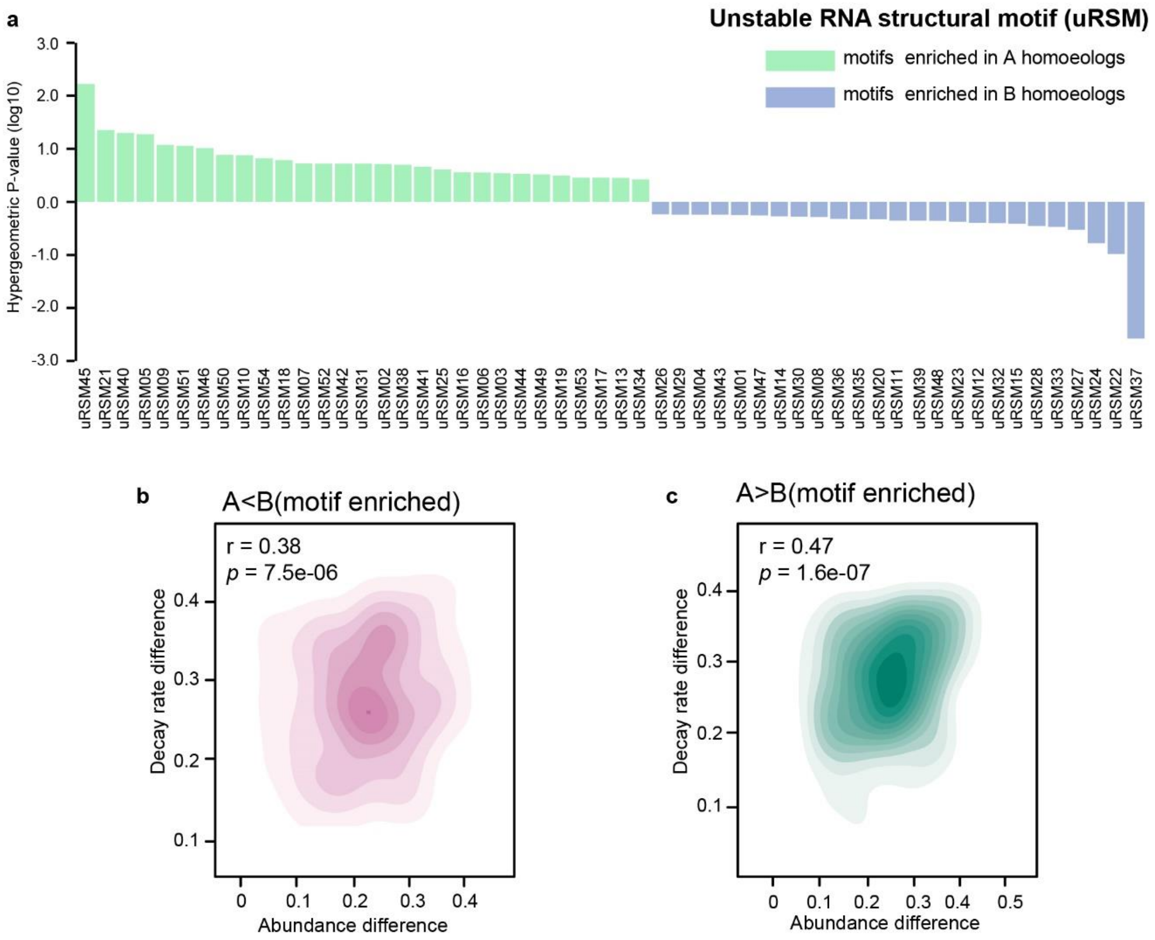

Supplementary Fig. 8 | The subgenomes of wheat exhibit distinct preferences for the unstable RNA structural motifs and the correlations between the differential RNA decay rates and the differential steady-state RNA levels. a, The different enrichments of the unstable RNA structural motifs (uRSMs) in the A and B subgenomes. **b-c,** The subgenomic preferences for stability-associated RNA structural motifs contribute to the subgenomic asymmetry of gene expression. The correlations between the differential RNA decay rates and the differential steady-state RNA levels in the homoeologous gene pairs possessing stability-associated RNA structural motifs (decay rate, A < B, r = 0.38, p = 7.5e-6; A > B, r = 0.47, p = 1.6e-6, two-sided Pearson correlation test).

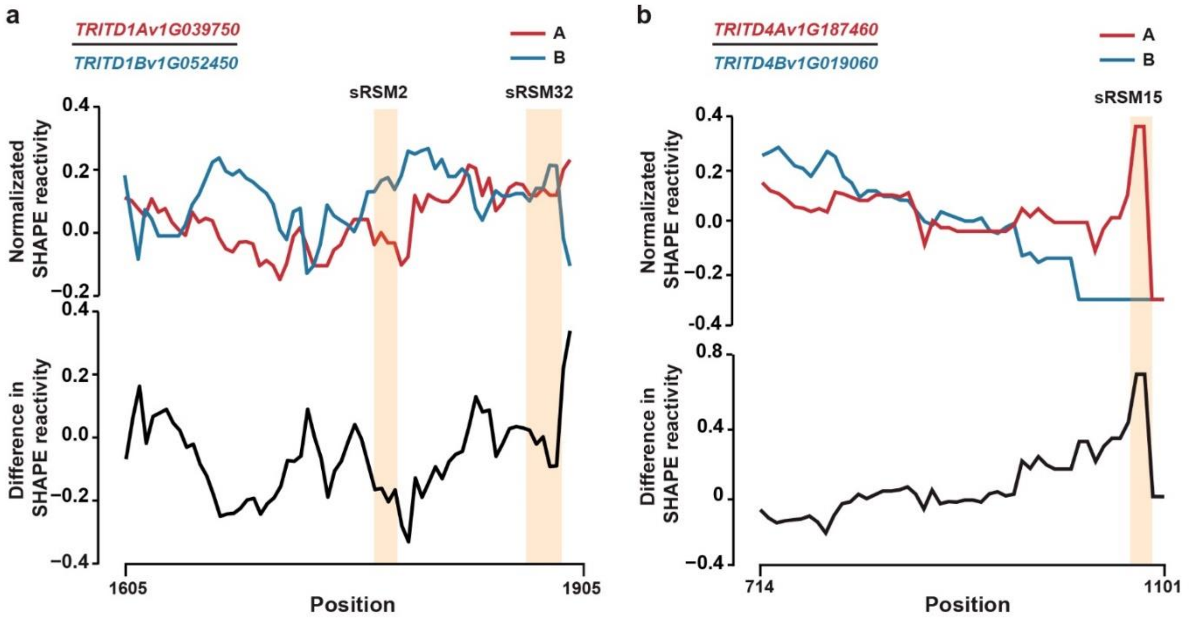

Supplementary Fig. 9 | SHAPE reactivity profiles (top) and SHAPE reactivity differences (bottom) for the 3’ UTRs of homoeologous gene pairs. a, *TRITD1Av1G039750* and *TRITD1Bv1G052450*. **b,** *TRITD4Av1G187460* and *TRITD4Bv1G019060*. Stable RNA structural motifs are highlighted in orange. The A and B subgenomes are aligned based on sequence alignment. The positions in the plot correspond to the positions of the A subgenome.

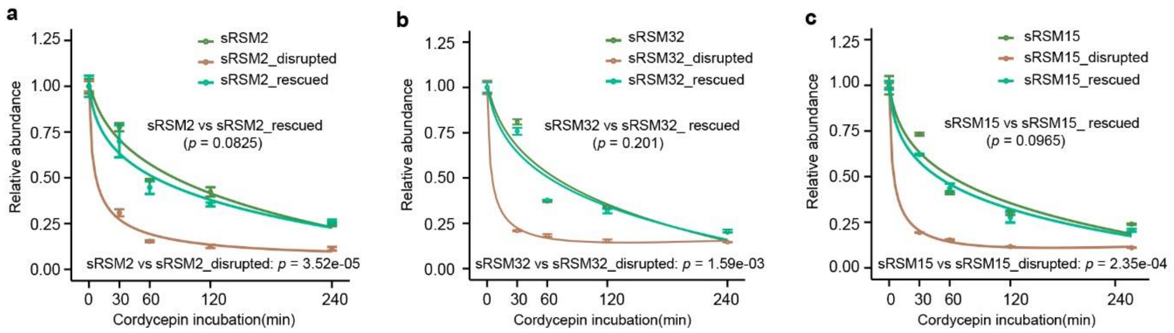

Supplementary Fig. 10 | Decay rates of constructs with original, rescued, or disrupted motif structures across the sRSMs. The dark green line represents the decay trend for sRSM constructs with original motif structures, while the light green line represents the decay trend for sRSM constructs with motif structures rescued by redesigned sequence. While the brown line indicates the decay trend for constructs with motif structures disrupted across all motifs. **a,** sRSM2 **(**error bars indicating ±SEM, $n_{replicate\ samples}$ = 3, sRSM2 vs sRSM2_disrupted: p = 3.52e-05, sRSM2 vs sRSM2_rescued: p = 0.0825, one-sided repeated measures ANOVA test); **b,** sRSM32 (error bars indicating ±SEM, $n_{replicate\ samples}$ = 3, sRSM32 vs sRSM32_disrupted: p = 1.59e-03, sRSM32 vs sRSM32_ rescued: p = 0.201, one-sided repeated measures ANOVA test); **c,** sRSM15 (error bars indicating ±SEM, $n_{replicate\ samples}$ = 3, sRSM15 vs sRSM15_disrupted: p = 2.35e-04, sRSM15 vs sRSM15_ rescued: p = 0.0965, one-sided repeated measures ANOVA test).

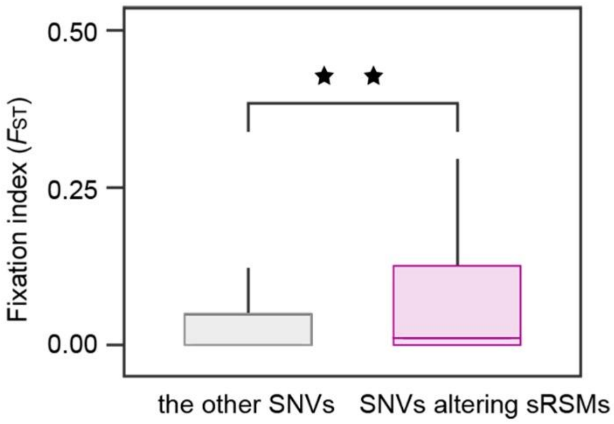

Supplementary Fig. 11 | SNVs that alter subgenomic preferred sRSMs were selected during domestication. Boxplot showing the fixation index (F_{ST}) of SNVs within stable RNA structural motifs regions and other SNVs across wheat accessions during domestication (**p < 0.01, one-sided Student's *t*-test).

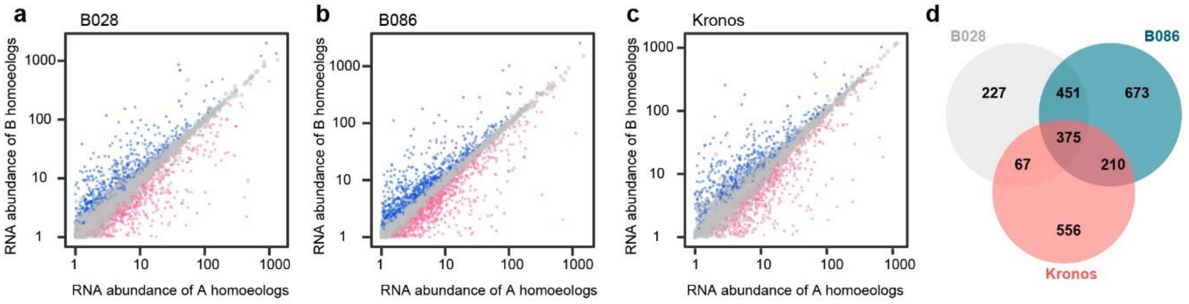

Supplementary Fig. 12 | Analysis of subgenomic differences in steady-state mRNA abundances among wheat varieties. a-c, Scatter plots showing differentially expressed homoeologous gene pairs between A and B subgenomes in B028 (WEW) **(a)**, B086 (DEW) **(b)**, and Kronos (DW) **(c)** ($p < 0.05$, one-sided Student's *t*-test). **d,** Venn diagram illustrating the overlap of 375 common differentially expressed gene pairs across all three varieties.

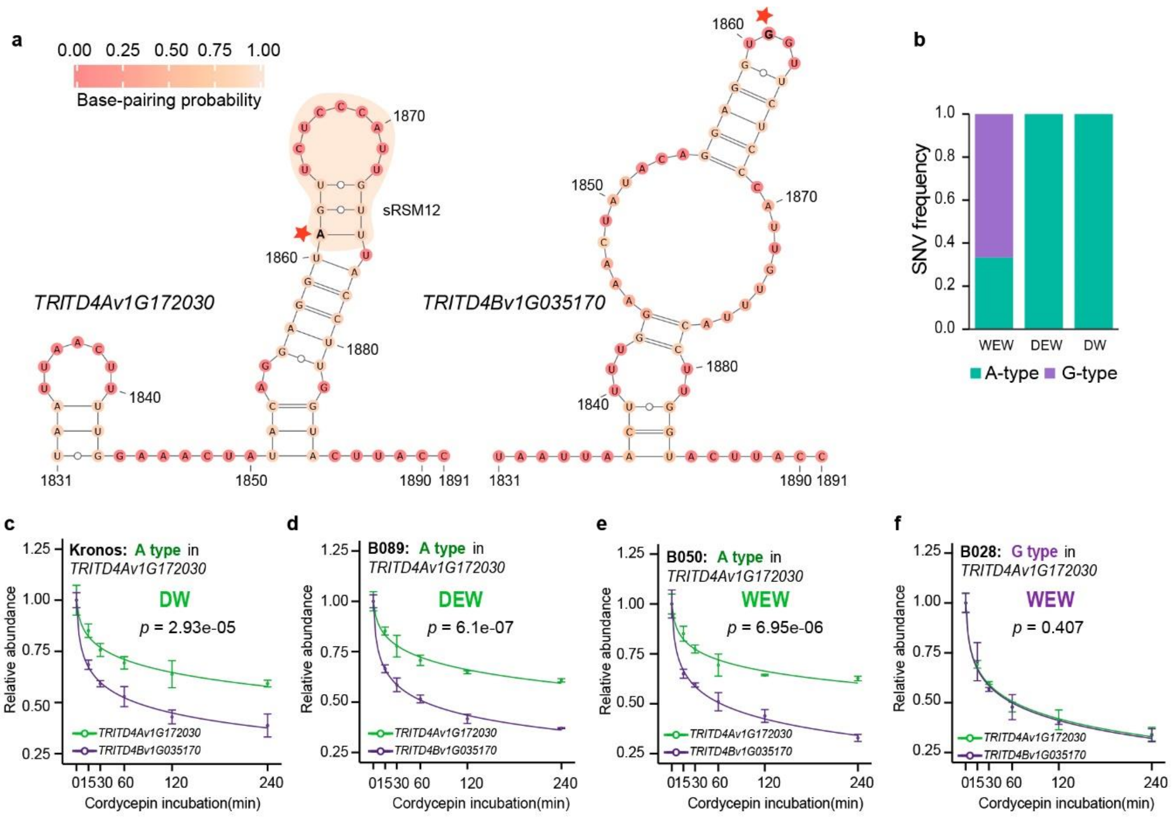

Supplementary Fig. 13 | The stability-associated RNA structural motif in *TRITD4Av1G172030* may have evolved during wheat domestication. a, Diagram of the SNV, A1861, within sRSM12 in the 3’ UTR of *TRITD4Av1G172030* (marked with red asterisks). This A-type nucleotide is replaced by a G in the 3' UTR of *TRITD4Bv1G035170*, disrupting the sRSM12 structure. **b,** The distribution of A- and G-type nucleotides in *TRITD4Av1G172030* across different wheat accessions as reported[2]. **c-f,** qRT-PCR validation showing the decay trends of A- and G-type mRNAs of *TRITD4Av1G172030* across DW, DEW, and WEW accessions (error bars indicating ±SEM, $n_{replicate\ samples}$= 3, **c:** p = 2.93e-05, **d:** p = 6.1e-07, **e:** p = 6.95e-06, **f:** p = 0.407, one-sided repeated measures ANOVA test).

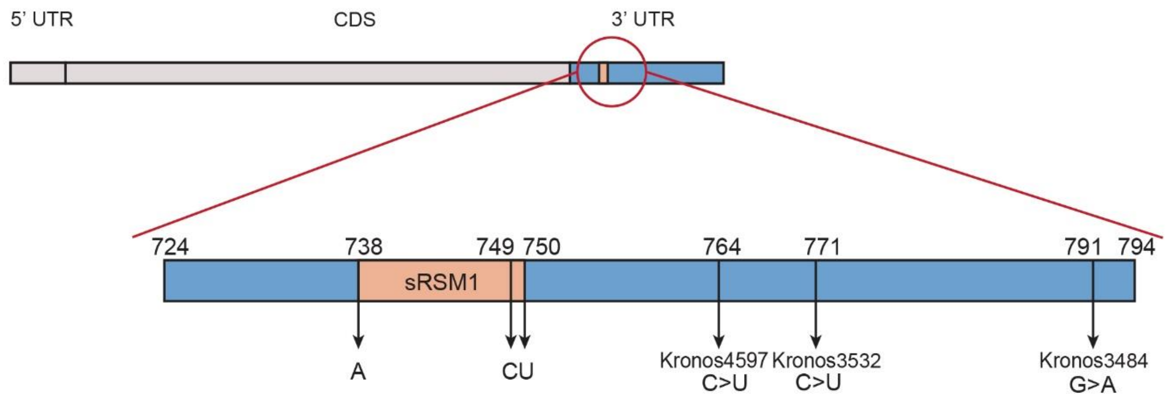

Supplementary Fig. 14 | Positional information of Kronos EMS mutant lines within the 3' UTR of *TRITD4Av1G006890*. This diagram illustrates the three EMS-mutagenized mutants within the 3' UTR of *TRITD4Av1G006890*: Kronos4597, Kronos3532, and Kronos3484. Notably, the mutation in Kronos3532 disrupts a G-C base pair close to the sRSM1 site while the mutations on Kronos4597 and Kronos3484 were localized on the single-stranded sites.
