# Supplementary material for: Unveiling RNA structure-mediated regulations of RNA stability in wheat
Source: Nat Commun. 2024 Nov 20;15:10042. doi: 10.1038/s41467-024-54172-7 (PMC11579497; doi:10.1038/s41467-024-54172-7)
Supplement: Supplementary file 3 — Description of Additional Supplementary Files [file 41467_2024_54172_MOESM3_ESM.pdf]

Description of Additional Supplementary Files

Supplementary Data 1: Reads counts and mapping rates for each library at seven time points

Supplementary Data 2: mRNA decay rates and half-lives

Supplementary Data 3: Information on intronless genes and details about the genes targeted by miRNA

Supplementary Data 4: Genes with both high translation efficiency and high decay rates

Supplementary Data 5: Several mRNA features contribute to the differences in stability between homoeologous gene pairs

Supplementary Data 6: 64 RNA structural stable motifs and 54 RNA structural unstable motifs

Supplementary Data 7: Sequence information of the synthesized short fragments of the three sRSMs and primers

Supplementary Data 8: mRNA decay factors for normalization
